# Algorithmic Information Distortions in Node-Aligned and Node-Unaligned Multidimensional Networks

**DOI:** 10.3390/e23070835

**Published:** 2021-06-29

**Authors:** Felipe S. Abrahão, Klaus Wehmuth, Hector Zenil, Artur Ziviani

**Affiliations:** 1 National Laboratory for Scientific Computing (LNCC), Petropolis 25651-075, RJ, Brazil; wehmuthklaus@gmail.com (K.W.); ziviani@lncc.br (A.Z.); 2 Laboratoire de Recherche Scientifique (LABORES) for the Natural and Digital Sciences, Algorithmic Nature Group, 75005 Paris, France; hector.zenil@cs.ox.ac.uk; 3 British Library, The Alan Turing Institute, 2QR, 96 Euston Rd, London NW1 2DB, UK; 4 Oxford Immune Algorithmics, Reading RG1 3EU, UK; 5 Center for Molecular Medicine, Algorithmic Dynamics Lab, Unit of Computational Medicine, Department of Medicine Solna, Karolinska Institute, SE-171 77 Stockholm, Sweden

**Keywords:** multidimensional networks, network complexity, lossless compression, information distortion, graph isomorphism, multiaspect graphs, multilayer networks, information content analysis, algorithmic complexity

## Abstract

In this article, we investigate limitations of importing methods based on algorithmic information theory from monoplex networks into multidimensional networks (such as multilayer networks) that have a large number of extra dimensions (i.e., aspects). In the worst-case scenario, it has been previously shown that node-aligned multidimensional networks with non-uniform multidimensional spaces can display exponentially larger algorithmic information (or lossless compressibility) distortions with respect to their isomorphic monoplex networks, so that these distortions grow at least linearly with the number of extra dimensions. In the present article, we demonstrate that node-unaligned multidimensional networks, either with uniform or non-uniform multidimensional spaces, can also display exponentially larger algorithmic information distortions with respect to their isomorphic monoplex networks. However, unlike the node-aligned non-uniform case studied in previous work, these distortions in the node-unaligned case grow at least exponentially with the number of extra dimensions. On the other hand, for node-aligned multidimensional networks with uniform multidimensional spaces, we demonstrate that any distortion can only grow up to a logarithmic order of the number of extra dimensions. Thus, these results establish that isomorphisms between finite multidimensional networks and finite monoplex networks do not preserve algorithmic information in general and highlight that the algorithmic information of the multidimensional space itself needs to be taken into account in multidimensional network complexity analysis.

## 1. Introduction

Algorithmic information theory (AIT) [1,2,3,4] has been playing an important role in the investigation of network complexity. More comprehensive surveys of previous work regarding algorithmic information (or lossless compression) of networks or graphs, together with comparisons to entropy-based methods, can be found in [5,6,7]. For example, AIT presented contributions to the challenge of causality discovery in network modeling [8], network summarization [9,10], automorphism group size [11], network topological properties [11,12], and the principle of maximum entropy and network topology reprogrammability [13]. Beyond monoplex networks (or graphs), Santoro and Nicosia [14] investigated the reducibility problem of multiplex networks. As the study of multidimensional networks, such as multilayer networks and dynamic multilayer networks, has become one of the central topics in network science, further exploration of algorithmic information has become relevant.

In this sense, we show that the currently existing methods that are based on AIT applied to monoplex networks (or graphs) cannot be straightforwardly imported into the multidimensional case without a proper evaluation of the algorithmic information distortions that might be present. Our results show the importance of multidimensional network encodings into which the necessary information for determining the multidimensional space itself is also embedded.

This article explores the possible combinations of node alignment and uniformity that can generate algorithmic information distortions and establishes worst-case error margins for these distortions in multidimensional network complexity analyses. We present a theoretical investigation of *worst-case* algorithmic information content (or lossless compressibility) distortions in four types of multidimensional networks that have sufficiently large multidimensional spaces. We show that:

1. *node-unaligned* multidimensional networks with *non-uniform* multidimensional spaces display exponentially larger distortions with respect to their respective isomorphic monoplex networks and that these worst-case distortions in the node-unaligned non-uniform case grow at least exponentially with the number of extra node dimensions;
2. *node-unaligned* multidimensional networks with *uniform* multidimensional spaces also display exponentially larger distortions with respect to their respective isomorphic monoplex networks and that these worst-case distortions in the node-unaligned uniform case also grow at least exponentially with the number of extra node dimensions;
3. *node-aligned* multidimensional networks with *non-uniform* multidimensional spaces also display exponentially larger distortions with respect to their respective isomorphic monoplex networks, but these worst-case distortions in the node-aligned non-uniform grow at least linearly with the number of extra node dimensions;
4. *node-aligned* multidimensional networks with *uniform* multidimensional spaces can *only* display distortions up to a logarithmic order of the number of extra node dimensions.

These four results are demonstrated in our final Theorem 5 by combining previous results from [15] (which are briefly remembered in Section 2) and new results from Section 4 in this article. We highlight that, unlike the node-aligned non-uniform case studied in [15] (in which the worst-case distortions were shown to grow at least linearly with the number of extra node dimensions), we demonstrate that the worst-case distortions in the node-unaligned cases grow at least exponentially with the number of extra node dimensions. In addition, we highlight that the node-aligned uniform case is shown to be the one in which the algorithmic information content of any multidimensional network and the algorithmic information content of its isomorphic monoplex network is proved to be much less distorted as the number of node dimensions increases. This occurs because any algorithmic information distortion can only grow up to a logarithmic order of the number of extra node dimensions, which contrasts with the exponential growth in the node-unaligned cases and with the linear growth in the node-aligned non-uniform case.

The remainder of the article is organized as follows: In Section 2, we present the previous results achieved in [15], which correspond to the node-aligned non-uniform case. In Section 3, we study basic properties of encoded node-unaligned multidimensional networks. In Section 4, we demonstrate the new mathematical results. In Section 5, we discuss the limitations and conditions for importing monoplex network complexity to multidimensional network algorithmic information. Section 6 concludes the paper.

This article is an extended version of a previous conference paper [15], whose results correspond to the node-aligned non-uniform case presented in Section 2. A preprint version of the present article containing additional proofs is available at [16].

## 2. Previous Work: The Node-Aligned Non-Uniform Case

Theorem 1 and Corollary 1 below restate the results in [15], which show that, although for every *node-aligned* multidimensional network there is a monoplex network that is isomorphic to this multidimensional network [17], they are not always equivalent in terms of algorithmic information. In addition, they demonstrate that these distorted values of algorithmic information content grow at least linearly with the value of *p* (i.e., number of extra node dimensions).

**Theorem** **1.** *There are arbitrarily large encodable simple node-aligned MAGs $G_{c}$ given $\tau (G_{c})$ with arbitrarily large non-uniform multidimensional spaces such that*

$$K\left(\langle \tau (G_{c})\rangle \right)+O\left(1\right)\geq K\left(\langle E(G_{c})\rangle \;|\;x\right)\geq K\left(\langle \tau (G_{c})\rangle \right)-O\left(\log_{2}\left(K(\langle \tau (G_{c})\rangle )\right)\right),$$

*with $K\left(\langle E(G_{c})\rangle \right)\geq p-O\left(1\right)$ and $K\left(x\right)=O\left(\log_{2}\left(p\right)\right)$, where x is the respective (node-aligned) characteristic string and p is the order of the MAG $G_{c}$.*

**Corollary** **1.** *There are an infinite family $F_{1}$ of simple (node-aligned) MAGs and an infinite family $F_{2}$ of classical graphs, where every classical graph in $F_{2}$ is MAG-graph-isomorphic to at least one MAG in $F_{1}$, such that, for every constant c∈N, there are $G_{c}\in F_{1}$ and a $G_{G_{c}}\in F_{2}$ that is MAG-graph-isomorphic to $G_{c}$, where*

$$O\left(\log_{2}\left(K(\langle E(G_{c})\rangle )\right)\right)>c+K\left(\langle E\left(G_{G_{c}}\right)\rangle \right).$$

Remember from [15] that the *multidimensional networks* are mathematically represented by *multiaspect graphs* (MAGs) $G=\left(A,E\right)$ [17,18], where A is the list *A* of aspects and E is the composite edge set (i.e., the set of existent composite edges). This way, note that $A=\left(A(G)[1],\cdots ,A(G)[i],\cdots ,A(G)[p]\right)$ is a list of sets, where each set A(G)[i] in this list is an *aspect* (or node dimension [19]). (In this article one can employ the terms “aspect” or “node dimension” interchangeably). The *companion tuple* of a MAG G is denoted by τ(G) [18], where

$$\tau \left(G\right)=\left(|A(G)[1]|,\cdots ,|A(G)[p]|\right)$$

and *p* is called the *order* of the MAG. $\langle \tau (G_{c})\rangle$ denotes an arbitrary encoded form of the companion tuple. We refer to the discrete *multidimensional space* of a MAG as the discrete cartesian product ${✕}_{i=2}^{p}A\left(G\right)\left[i\right]$ into which the nodes of the network are embedded. In the particular case A(G)[i]=A(G)[j] holds for every i,j≤p, we say the multidimensional space of the MAG is *uniform*.

A MAG is said to be *node aligned iff* $V\left(G\right)={✕}_{i=1}^{p}A\left(G\right)\left[i\right]$ and E(G)=V(G)✕V(G), where V(G) denotes the node-aligned set of all *composite vertices*$v=(a_{1},\cdots ,a_{p})$ of G, and E(G) denotes the set of all possible *composite edges* $e=(\left(a_{1},\cdots ,a_{p}\right),\left(b_{1},\cdots ,b_{p}\right))$ of G.

Following the common notation and nomenclature [20,21,22], G=(V,E) denotes a general (directed or undirected) *graph*, where *V* is the finite set of vertices and E⊆V×V; if a graph only contains undirected edges and does not contain self-loops, then it is called a *simple* graph. A graph *G* is said to be (vertex-)labeled when the members of *V* are distinguished from one another by labels such as $v_{1},v_{2},\cdots ,v_{\left|V\right|}$. If a simple graph is labeled this way by natural numbers, that is, V={1,⋯,n} with n∈N, then it is called a *classical* graph. In a direct analogy to classical graphs, a *simple* MAG $G_{c}=\left(A,E\right)$ is an *undirected* MAG without *self-loops*, so that the set $E_{c}$ of all possible undirected and non-self-loop composite edges is defined by $E_{c}\left(G_{c}\right):=\left\{\left\{u,v\right\}|u,v\in V\left(G_{c}\right)\right\}$ and $E\left(G_{c}\right)\subseteq E_{c}\left(G_{c}\right)$ always holds. Hence, if a *simple* MAG $G_{c}$ is (composite-vertex-)labeled with natural numbers (i.e., for every i≤p, $A\left(G\right)\left[i\right]=\left\{1,\cdots ,\left|A(G)[i]\right|\right\}\subset N$), then we say that $G_{c}$ is a *classical* MAG. Note that, for classical MAGs, the companion tuples completely determine the discrete *multidimensional space* of the respective MAGs. For the present purposes of this article, all graphs *G* will be classical graphs and all MAGs will be simple MAGs (whether node-aligned or node-unaligned). Following the usual definition of encodings, a MAG is *encodable given* $\tau (G_{c})$ *iff* there is a fixed program that, given $\tau (G_{c})$ as input, can univocally encode any possible $E\left(G_{c}\right)$ that shares the same companion tuple. In other words, if the companion tuple $\tau (G_{c})$ of the MAG $G_{c}$ is already known, then one can computably retrieve the position of any composite edge e={u,v} in the chosen data representation of $G_{c}$ from both composites vertices u and v, and vice-versa.

$\langle E(G_{c})\rangle$ denotes the *(node-aligned) composite edge set string* $\langle \langle e_{1},z_{1}\rangle ,\cdots ,\langle e_{n},z_{n}\rangle \rangle$ such that $z_{i}=1$ *iff* $e_{i}\in E\left(G_{c}\right)\;,$ where $z_{i}\in \left\{0,1\right\}$ with $1\leq i\leq n=|E_{c}\left(G_{c}\right)|$. Note that a composite edge set string is an encoding of a composite edge list, which in turn is a generalization of edge lists [23] so as to deal with the encoding of a MAGs instead of graphs. Thus, the reader may also interchangeably call the composite edge set string by *composite edge list encoding*. A bit string *x* of length $\left|E_{c}\left(G_{c}\right)\right|$ is said to be a *(node-aligned) characteristic string* of $G_{c}$ *iff* there is a fixed program that, given *x* as input, computes the composite edge set $E(G_{c})$ and there is another fixed program that, given the encoded composite edge set $E(G_{c})$ as input, returns the string *x*. Note that the characteristic string of a MAG differs from the composite edge set string by the fact that an encoding of the companion tuple is already embedded into the composite edge set string. On the other hand, Theorem 1 shows this is not always the case for characteristic strings, which in some cases do not have sufficient information for determining their respective companion tuples.

As established in [17], we say a (node-aligned) MAG G is *MAG-graph-isomorphic* to a graph *G* *iff* there is a bijective function f:V(G)→V(G) such that e∈E(G) *iff* $(f\left(\pi_{o}\left(e\right)\right),f\left(\pi_{d}\left(e\right)\right))\in E\left(G\right)\;,$ where $\pi_{o}$ is a function that returns the origin composite vertex of a composite edge and $\pi_{d}$ is a function that returns the destination composite vertex of a composite edge. This way, $G_{G_{c}}$ denotes the classical graph which is MAG-graph isomorphic to the simple MAG $G_{c}$. We know this classical graph always exists from [17]. In order to avoid ambiguities in the nomenclature with the classical isomorphism in graphs (which is usually a vertex label transformation) we call: such an isomorphism between a MAG and graph from [17] a *MAG-graph isomorphism*; the usual isomorphism between graphs [20,21] as *graph isomorphism*; and the isomorphism between two MAGs G and $G^{\prime }$ as *MAG isomorphism*.

K(w) denotes the (unconditional) *prefix algorithmic complexity*, which is the length $l(w^{*})$ of the shortest program $w^{*}$ such that $U(w^{*})=w$. The conditional prefix algorithmic complexity is denoted by $K\left(\cdot |\cdot \right)$.

More detailed notation, nomenclature and properties of encodable node-aligned simple MAGs can be found in [15,16].

### Beyond the Node-Aligned Case Studied in Previous Work

The results in [15] demonstrate that *node-aligned* multidimensional networks with *non-uniform* multidimensional spaces can display exponentially larger algorithmic information distortions with respect to their isomorphic monoplex networks. On the other hand, one may want to also investigate the extent of those distortions when the multidimensional network is *node unaligned* and/or the multidimensional space is *uniform*. The results we will demonstrate in the following sections differ from the ones of the present Section 2 (and from [15]) by investigating algorithmic information distortions in *node-unaligned* multidimensional networks either with *uniform* or *non-uniform* multidimensional spaces. Indeed, in the forthcoming node-unaligned case, additional algorithmic information may be necessary in order to determine to which permutation $\alpha \in {✕}_{i=2}^{p}A\left(G\right)\left[i\right]$ a node does not belong, given that only the necessary and sufficient information to compute E (or $E_{M}$) is known *a priori*. We shall see that this leads to worst-case algorithmic information distortions that grow at least *exponentially* with the value of *p*, which differs from the *linear* growth presented by Theorem 1 from [15].

## 3. The Node-Unaligned Cases

With the purpose of addressing other variations of multidimensional networks in which a node not belonging to a certain $\alpha \in {✕}_{i=2}^{p}A\left(G\right)\left[i\right]$ has an important physical meaning, the node alignment can be relaxed. This gives rise to multidimensional networks that are not node aligned, such as node-unaligned multilayer networks [24] or node-unaligned multiplex networks [25].

As a multiplex network is usually understood as a particular case of a multilayer network [24,26,27] in which there is only one extra node dimension (i.e., d=1), we may focus only on a mathematical formulation of multilayer networks that allows nodes to be not aligned, which is given by $M=(V_{M},E_{M},V,L)$ [24], where:

1. *V* denotes the set of all possible vertices *v*;
2. $L={\left\{L_{a}\right\}}_{a=1}^{d}$ denotes a collection of d∈N sets $L_{a}$ composed of *elementary layers*$\alpha \in L_{a}$;
3. $V_{M}\subseteq V\times L_{1}\times \cdots \times L_{d}$ denotes the subset of all possible vertices paired to elements of $L_{1}\times \cdots \times L_{d}$;
4. $E_{M}\subseteq V_{M}\times V_{M}$ denotes the set of *interlayer* and/or *intralayer* edges connecting two *node-layer tuples*$\left(v,\alpha_{1},\cdots ,\alpha_{d}\right)\in V_{M}$.

In this regard, a multilayer network *M* is said to be *node-aligned* *iff* $V_{M}=V\times L_{1}\times \cdots \times L_{d}$. In the case each $\alpha \in {✕}_{i=2}^{p}A\left(G\right)\left[i\right]$ can be interpreted as (or is representing) a layer, it is important to note that there are some immediate equivalences between G and *M* [19]:

- *V* is the usual set of vertices, where V(G)≡A(G)[1];
- each set $L_{a}$ is the (a−1)-th aspect A(G)[a−1] of a MAG G;
- $V_{M}$ is a subset of the set V(G) of all composite vertices, where every node-layer tuple $\left(v,\alpha_{1},\cdots ,\alpha_{d}\right)\in V_{M}$ is a composite vertex v∈V(G);
- $E_{M}\subseteq E\left(G\right)$ is a subset of the set of all composite edges (u,v) for which $u,v\in V_{M}$.

Thus, if $V_{M}=V\times L_{1}\times \cdots \times L_{d}$, then one will have that $V_{M}\equiv V\left(G\right)$ and $E_{M}\equiv E\left(G\right)$. This way, besides notation distinctions, it directly follows that a node-aligned multilayer network *M* is totally equivalent to a MAG G; and, therefore, every result in this paper holding for simple node-aligned MAGs $G_{c}$ automatically holds for node-aligned multilayer networks *M* that only have undirected edges and do not contain self-loops. Nevertheless, since the algorithmic information distortions are based on (and are limited by) the algorithmic information of the multidimensional space itself and the uniformity (or non uniformity) of the multidimensional space is determined by the aspects, we highlight that representing multidimensional networks with MAGs, aspects and companion tuples facilitates the achievement of our theoretical results.

With the purpose of extending our results to the node-unaligned case, we need to introduce a variation of MAGs so as to allow into the mathematical formalization the possibility of some vertices not being paired with some α’s, where $\alpha \in {✕}_{i=2}^{p}A\left(G\right)\left[i\right]$. Moreover, we need that node-aligned MAGs become particular cases of our new formalization such that the algorithmic information between the two formalizations becomes equivalent when the MAG is node-aligned, which we will show in Lemma 3. To this end, we introduce a modification on the definition of MAG so that the major differences are in the set of composite vertices and, consequentially, in the set of composite edges.

**Definition** **1.** *We define a node-unaligned MAG as a triple $G_{ua}=\left(A,V_{ua},E_{ua}\right)$ in which*

$$A=\left(A\left(G_{ua}\right)\left[1\right],\cdots ,A\left(G_{ua}\right)\left[i\right],\cdots ,A\left(G_{ua}\right)\left[p\right]\right)$$

*is a list of sets (each of each is an aspect of $G_{ua}$),*

$$V_{ua}\left(G_{ua}\right)\subseteq V\left(G_{ua}\right)=\overset{p}{\underset{i=1}{✕}}A\left(G_{ua}\right)\left[i\right]$$

*is the set of existing composite vertices, and*

$$E_{ua}\subseteq E_{ua}\left(G_{ua}\right)=V_{ua}\left(G_{ua}\right)\times V_{ua}\left(G_{ua}\right)$$

*is the set of present composite edges $\left(u,v\right)$.*

The definition of simple node-unaligned MAGs $G_{uac}=\left(A,V_{ua},E_{ua}\right)$ follows analogously to the aligned case by just restricting the set of all possible composite edges, so that $E_{uac}\left(G_{uac}\right):=\left\{\left\{u,v\right\}|u,v\in V_{ua}\left(G_{uac}\right)\right\}$ and $E_{ua}\left(G_{uac}\right)\subseteq E_{uac}\left(G_{uac}\right)$ hold. In addition, all other terminology of *order*, *multidimensional space*, *uniformity*, *(composite-vertex-)labeling*, and *classical MAGs* apply analogously as in Section 2 (see also [16]).

As in the node-aligned case, in order to define an unaligned version for the companion tuple, the latter should completely determine the size of the set $E_{ua}\left(G_{ua}\right)$ and, if $G_{ua}$ is a classical MAG, then the companion tuple should completely determine the multidimenional space of $G_{ua}$. In this sense, a node-unaligned version of the companion tuple also needs to carry the necessary and sufficient information for computably retrieving the set $V_{ua}\left(G_{ua}\right)$.

**Definition** **2.** *The node-unaligned companion tuple $\tau_{ua}$ of a MAG $G_{ua}$ is defined by the pair of tuples*

$$\tau_{ua}\left(G_{ua}\right):=\left(\left(|A\left(G_{ua}\right)\left[1\right]|,\cdots ,|A\left(G_{ua}\right)\left[p\right]|\right),\left(m_{1},\cdots ,m_{\left|V\left(G_{ua}\right)\right|}\right)\right)\;,$$

*such that, for every $v_{i}\in V\left(G_{ua}\right)$ in a previously chosen arbitrary computable enumeration of $V\left(G_{ua}\right)$, $v_{i}\in V_{ua}\left(G_{ua}\right)$ iff $m_{i}=1\;,$ where $m_{i}\in \left\{0,1\right\}$ and $1\leq i\leq \left|V\left(G_{ua}\right)\right|$.*

As for encoding $\tau_{ua}$, one can also employ the recursive pairing function $\langle \cdot ,\cdot \rangle$ as usual:

$$\langle \tau_{ua}\left(G_{ua}\right)\rangle :=\langle \langle \left|A\left(G_{ua}\right)\left[1\right]\right|,\cdots ,\left|A\left(G_{ua}\right)\left[p\right]\right|\rangle ,\langle m_{1},\cdots ,m_{\left|V\left(G_{ua}\right)\right|}\rangle \rangle \;.$$

The MAG-graph isomorphism also suffers a slight modification:

**Definition** **3.** *$G_{ua}$ is unaligning MAG-graph-isomorphic to a graph G iff there is a bijective function $f:V_{ua}\left(G_{ua}\right)\to V\left(G\right)$ such that $e\in E_{ua}\left(G_{ua}\right)\subseteq E_{ua}\left(G_{ua}\right)$ iff $(f\left(\pi_{o}\left(e\right)\right),f\left(\pi_{d}\left(e\right)\right))\in E\left(G\right)\;,$ where $\pi_{o}$ is a function that returns the origin composite vertex of a composite edge and $\pi_{d}$ is a function that returns the destination composite vertex of a composite edge.*

This way, we can straightforwardly obtain the following Theorem 2 analogously to the proof of (Theorem 1, p. 54, [17]).

**Theorem** **2.** *For every MAG $G_{ua}$ of order p>0, where all aspects are non-empty sets, there is a unique (up to a graph isomorphism) graph $G_{G_{ua}}^{ua}=\left(V,E\right)$ that is unaligning MAG-graph-isomorphic to $G_{ua}$, where*

$$|V\left(G_{G_{ua}}^{ua}\right)|=\left|V_{ua}\left(G_{ua}\right)\right|\;.$$

The proof of Theorem 2 can be found in [16].

It is important to note our choice of notation distinction between the node-aligned and the node-unaligned case when a graph is MAG-graph-isomorphic to a MAG. The graph $G_{G_{ua}}$ is said to be *aligning* MAG-graph-isomorphic to the MAG when the set of possible composite vertices is complete, that is, when it is taken from $V(G_{ua})$. This was the case in Section 2. On the other hand, the graph $G_{G_{ua}}^{ua}$ is said to be *unaligning* MAG-graph-isomorphic to the MAG (which is the case of Definition 3, Theorem 2, Corollary 2, and Theorem 5(I)(b)) when the possible composite vertices are taken from $V_{ua}\left(G_{ua}\right)$ instead of $V(G_{ua})$.

### Encoding Node-Unaligned Multiaspect Graphs

Encodability of node-unaligned MAGs given the companion tuple works in the same way as in the node-aligned case. That is, a node-unaligned simple MAG $G_{uac}$ is *encodable given* $\tau_{ua}\left(G_{uac}\right)$ *iff* there is a fixed program that, given $\langle \tau_{ua}\left(G_{uac}\right)\rangle$ as input, can univocally encode any possible $E_{ua}\left(G_{uac}\right)$ that shares the same (node-unaligned) companion tuple.

First, as in the node-aligned case, note that the encodability of *classical* node-unaligned MAGs given the companion tuple can be promptly proved to hold (the proof can be found in [16]):

**Lemma** **1.** *Any arbitrary node-unaligned classical MAG $G_{uac}$ is encodable given $\tau_{ua}\left(G_{uac}\right)$.*

Secondly, note that the characteristic string of a node-unaligned MAG is defined in a similar way as in [15] (see also Section 2):

**Definition** **4.** *Let $\left(e_{1},\cdots ,e_{\left|E_{uac}\left(G_{uac}\right)\right|}\right)$ be any arbitrary ordering of all possible composite edges between existing composite vertices of a node-unaligned simple MAG $G_{uac}$. We say that a bit string $x^{\prime }$ with $l\left(x^{\prime }\right)=\left|E_{uac}\left(G_{uac}\right)\right|$ is a node-unaligned characteristic string of $G_{uac}$ if, for every $e_{j}\in E_{uac}\left(G_{uac}\right)$, one has $e_{j}\in E_{ua}\left(G_{uac}\right)$ iff the j-th digit in x′ is 1, where $1\leq j\leq l(x^{\prime })$.*

Now, for the node-unaligned composite edge set string, the definition may seem not so straightforwardly translated from node-aligned case. As one can see below, it is based on a sequence of the $\left|E_{c}\left(G_{uac}\right)\right|$ composite edges, and not on the sequence of $\left|E_{uac}\left(G_{uac}\right)\right|$ composite edges. This is because one needs to embed into node-unaligned composite edge set strings not only the characteristic function of the set $E_{ua}\left(G_{uac}\right)$, as in the node-aligned case, but also the characteristic function of the set $V_{ua}\left(G_{uac}\right)$ (which becomes in turn determined by the $k_{i}$’s and $h_{i}$’s in the following definition):

**Definition** **5.** *Let $\left(e_{1},\cdots ,e_{\left|E_{c}\left(G_{uac}\right)\right|}\right)$ be any arbitrary ordering of all possible composite edges of a node-unaligned simple MAG $G_{uac}$. Then, $\langle E_{ua}\left(G_{uac}\right)\rangle$ denotes the node-unaligned composite edge set string $\langle \langle e_{1},z_{1}^{\prime },k_{1},h_{1}\rangle ,\cdots ,\langle e_{n},z_{n}^{\prime },k_{n},h_{n}\rangle \rangle$ such that:*

$$z_{i}^{\prime }=1\Leftrightarrow e_{i}\in E_{ua}\left(G_{uac}\right)\;,$$

$$k_{i}=1\Leftrightarrow \left(e_{i}=\left(v,u\right)\;\wedge \;v\in V_{ua}\left(G_{uac}\right)\right)$$

*and*

$$h_{i}=1\Leftrightarrow \left(e_{i}=\left(v,u\right)\;\wedge \;u\in V_{ua}\left(G_{uac}\right)\right)\;,$$

*where $z_{i}^{\prime },k_{i},h_{i}\in \left\{0,1\right\}$ with $1\leq i\leq n=|E_{c}\left(G_{uac}\right)|$.*

This way, we guarantee not only that the characteristic string $x^{\prime }$ can always be computably extracted from $\langle E_{ua}\left(G_{uac}\right)\rangle$, but also that the set $V_{ua}\left(G_{uac}\right)$ can be computably extracted from $\langle E_{ua}\left(G_{uac}\right)\rangle$. This will be important in the proof of Theorem 3 later on. Moreover, once the ordering of $E_{c}\left(G_{uac}\right)$ assumed in Definition 5 is preserved by the subsequence that exactly corresponds to the ordering of $E_{uac}\left(G_{uac}\right)$ assumed in Definition 4, we have in Lemma 2 below that both the node-unaligned simple MAG and its respective node-unaligned characteristic string become “equivalent” in terms of algorithmic information, but again (as occurred for the node-aligned case [15]) except for the minimum information necessary to encode the node-unaligned companion tuple:

**Lemma** **2.** *Let $x^{\prime }$ be a bit string. Let $G_{uac}$ be an encodable node-unaligned simple MAG given $\tau_{ua}\left(G_{uac}\right)$ such that $x^{\prime }$ is the respective node-unaligned characteristic string. Then,*

(1) $$\begin{array}{cc} K(\langle E_{ua}\left(G_{uac}\right)\rangle \;|\;x^{\prime }) & \leq K\left(\langle \tau_{ua}\left(G_{uac}\right)\rangle \right)+O\left(1\right) \end{array}$$

(2) $$\begin{array}{cc} K(x^{\prime }\;|\;\langle E_{ua}\left(G_{uac}\right)\rangle ) & \leq K\left(\langle \tau_{ua}\left(G_{uac}\right)\rangle \right)+O\left(1\right) \end{array}$$

(3) $$\begin{array}{cc} K(x^{\prime }) & =K\left(\langle E_{ua}\left(G_{uac}\right)\rangle \right)\pm O\left(K\left(\langle \tau_{ua}\left(G_{uac}\right)\rangle \right)\right). \end{array}$$

The proof of Lemma 2 can be found in [16].

As we saw in the node-aligned case, we shall see in the next section that node-unaligned characteristic strings are not in general equivalent to composite edge set strings $\langle E_{ua}\left(G_{uac}\right)\rangle$. More formally, we shall show in the following section that there are cases in which the algorithmic information necessary for retrieving the encoded form of the node-unaligned simple MAG from its node-unaligned characteristic string is close (except for a logarithmic term) to the upper bound given by Equation (1) in Lemma 2.

## 4. Worst-Case Algorithmic Information Distortions

In this section, we investigate worst-case algorithmic information distortions for *node-unaligned* MAGs when the multidimensional space is arbitrarily large. In particular, we study large multidimensional spaces that are *non-uniform* or *uniform*.

Before heading toward the theorems themselves, it is important to show the two cases in Lemmas 6 or 7 for which the set $V_{ua}\left(G_{uac}\right)$ trivializes the problem either by reducing it back to the node-aligned case or by reducing it to a problem of just inserting empty nodes.

The first trivializing case guarantees the consistency of our definitions of node-aligned and node-unaligned MAGs:

**Lemma** **3.** *Let $G_{uac}$ be a node-unaligned simple MAG with $V_{ua}\left(G_{uac}\right)=V\left(G_{uac}\right)$, where x is its node-aligned characteristic string and $x^{\prime }$ is its node-unaligned characteristic string. Then,*

(4) $$K\left(\langle E_{ua}\left(G_{uac}\right)\rangle \right)=K\left(\langle E(G_{uac})\rangle \right)\pm O\left(1\right)\;,$$

(5) $$K\left(x\right)=K\left(x^{\prime }\right)\pm O\left(1\right)\;,$$

*and*

(6) $$K\left(\langle \tau_{ua}\left(G_{uac}\right)\rangle \right)=K\left(\langle \tau (G_{uac})\rangle \right)\pm O\left(1\right)$$

*hold.*

The proof of Lemma 3 can be found in [16]. In fact, if $V_{ua}\left(G_{uac}\right)=V\left(G_{uac}\right)$, the same proof of Lemma 3 can be employed to show that the strings in the left side of the equations in Lemma 3 are respectively Turing equivalent to their counterparts in the right side. Therefore, for any $G_{uac}$ satisfying Lemma 3, any algorithmic information distortion occurs in the same manner as in the node-aligned case.

The second one guarantees the consistency between network connectedness and empty nodes. An *empty node* [24] is a totally unconnected node that is added to the network in order to recover the node alignment of a former node-unaligned network. Thus, as expected, if all the composite vertices in $V_{ua}\left(G_{uac}\right)$ are connected to at least another composite vertex in $V_{ua}\left(G_{uac}\right)$, then all the possible unconnected composite vertices are those that redundantly are empty nodes:

**Lemma** **4.** *Let $G_{uac}$ be a node-unaligned simple MAG in which every composite vertex in $V_{ua}\left(G_{uac}\right)$ is connected to at least another composite vertex in $V_{ua}\left(G_{uac}\right)$. Then,*

(7) $$K\left(\langle E_{ua}\left(G_{uac}\right)\rangle \right)=K\left(\langle E(G_{uac})\rangle \right)\pm O\left(1\right)$$

*holds and, additionally, $\langle E_{ua}\left(G_{uac}\right)\rangle$ is in fact Turing equivalent to $\langle E(G_{uac})\rangle$.*

The proof of Lemma 4 can be found in [16].

Note that in Lemma 4 one immediately has that $\langle E\left(G_{G_{uac}}^{ua}\right)\rangle$ can be computed from $\langle E\left(G_{G_{uac}}\right)\rangle$ with a simple algorithm that identifies totally unconnected vertices. Furthermore, one has that $\langle E\left(G_{G_{uac}}\right)\rangle$ can be computed from $\langle E\left(G_{G_{uac}}^{ua}\right)\rangle$, if the value of $\left|V\left(G_{uac}\right)\setminus V_{ua}\left(G_{uac}\right)\right|$ is also given as input. Therefore, for any MAG satisfying Lemma 4, the algorithmic information distortion between the MAG $G_{uac}$ and the *unaligning* MAG-graph-isomorphic classical graph $G_{G_{uac}}^{ua}$ can only differ from the algorithmic information distortion between the MAG $G_{uac}$ and the *aligning* MAG-graph-isomorphic classical graph $G_{G_{uac}}$ by $O\left(\log_{2}\left(|V\left(G_{uac}\right)\setminus V_{ua}\left(G_{uac}\right)|\right)\right)$ bits of algorithmic information.

Thus, as the reader might notice, the case in which the node nonalignment introduces more irreducible information into the composite edge set string is when $V_{ua}\left(G_{uac}\right)\neq V\left(G_{uac}\right)$ and not every unconnected composite vertex is empty. Under these conditions that demand a more careful theoretical analysis than the trivializing cases in Lemmas 3 or 4, we study now that exponential algorithmic information distortions can occur in the node-unaligned case. An extended version of the proof of Theorem 3 can be found in [16].

**Theorem** **3.** *There are encodable node-unaligned simple MAGs $G_{uac}$ given $\tau_{ua}\left(G_{uac}\right)$ with arbitrarily large non-uniform multidimensional spaces such that*

$$K\left(\langle \tau_{ua}\left(G_{uac}\right)\rangle \right)+O\left(1\right)\geq K\left(\langle E_{ua}\left(G_{uac}\right)\rangle \;|\;x^{\prime }\right)\geq K\left(\langle \tau_{ua}\left(G_{uac}\right)\rangle \right)-O\left(\log_{2}\left(K(\langle \tau_{ua}\left(G_{uac}\right)\rangle )\right)\right)$$

*with*

$$K\left(x^{\prime }\right)=O\left(p\right)=O\left(\log_{2}\left(K\left(\langle E_{ua}\left(G_{uac}\right)\rangle \right)\right)\right)\;,$$

*where $x^{\prime }$ is the respective node-unaligned characteristic string and p is the order of $G_{uac}$.*

**Proof.**  Let $G_{uac}$ be any node-unaligned simple MAG such that $w_{1}$ and $w_{2}$ are bit strings that, respectively, are finite initial segments of a 1-random real number y, where $l\left(w_{1}\right)=p$, $l\left(w_{2}\right)=\left|V\left(G_{uac}\right)\right|$, any $|A\left(G_{uac}\right)\left[i\right]|$ can only assume values in {1,2} in accordance to the bits of $w_{1}$, and the existence of a composite vertex in $V\left(G_{uac}\right)$ is determined by the bits of $w_{2}$. Remember that, if y is a *1-random real number* [4], then $K\left(y{↾}_{n}\right)\geq n-O\left(1\right)\;,$ where n∈N is arbitrary. From Lemma 1, we have that $G_{uac}$ is encodable given $\tau_{ua}\left(G_{uac}\right)$. Therefore, there is a program $q^{\prime }$ such that

(8) $$K\left(\langle \tau_{ua}\left(G_{uac}\right)\rangle \right)\leq l\left(\langle {\langle E_{ua}\left(G_{uac}\right)\rangle }^{*},q^{\prime }\rangle \right)\leq K\left(\langle E_{ua}\left(G_{uac}\right)\rangle \right)+O\left(1\right)$$

holds by the minimality of K(·) and by our construction of $q^{\prime }$. In addition, by our construction of $G_{uac}$, we will have that

(9) $$K\left(\langle \tau_{ua}\left(G_{uac}\right)\rangle \right)\leq p+\left|V\left(G_{uac}\right)\right|+O\left(\log_{2}\left(p\right)\right)$$

and, since y is 1-random,

(10) $$\left|V\left(G_{uac}\right)\right|-O\left(1\right)\leq K\left(\langle \tau_{ua}\left(G_{uac}\right)\rangle \right)+O\left(1\right)\;.$$

Since the exact number of 1’s appearing in $w_{2}$ is given by $\frac{\left|V\left(G_{uac}\right)\right|}{2}\pm o\left(\left|V\left(G_{uac}\right)\right|\right)\;,$ which follows from the Borel normality [3,28] of $w_{2}$, we will have that $K\left(l\left(x^{\prime }\right)\right)=O\left(p\right)$ holds from the fact that $l\left(x^{\prime }\right)=\left|E_{uac}\left(G_{uac}\right)\right|$. Moreover, the Borel normality of $w_{1}$ also guarantees that $\left|V\left(G_{uac}\right)\right|=2^{\left(\frac{p}{2}\pm o\left(p\right)\right)}$ and, as a consequence, $\Omega \left(p\right)=\log_{2}\left(\left|V\left(G_{uac}\right)\right|\right)\;.$ Now, since $E_{ua}\left(G_{uac}\right)$ and *p* were arbitrary, we can choose any node-unaligned characteristic string $x^{\prime }$ such that $K\left(x^{\prime }\;|\;l\left(x^{\prime }\right)\right)=O\left(1\right)$ holds and that there are some composite vertices in $V_{ua}\left(G_{uac}\right)$ that are not connected to any other composite vertex in $V_{ua}\left(G_{uac}\right)$. Thus, we will have that

(11) $$\begin{array}{cc} K\left(x^{\prime }\right)\; & \leq K\left(l\left(x^{\prime }\right)\right)+O\left(1\right)\leq \\ \; & \leq O\left(\log_{2}\left(K\left(\langle \tau_{ua}\left(G_{uac}\right)\rangle \right)\right)\right)\leq \\ \; & \leq O\left(\log_{2}\left(K\left(\langle E_{ua}\left(G_{uac}\right)\rangle \right)\right)\right) \end{array}$$

and

$$\begin{array}{cc} K\left(\langle \tau_{ua}\left(G_{uac}\right)\rangle \right)\; & \leq K\left(x^{\prime }\right)+K\left(\langle E_{ua}\left(G_{uac}\right)\rangle \;|\;x^{\prime }\right)+O\left(1\right)\leq \\ \; & \leq O\left(\log_{2}\left(K(\langle \tau_{ua}\left(G_{uac}\right)\rangle )\right)\right)+K\left(\langle E_{ua}\left(G_{uac}\right)\rangle \;|\;x^{\prime }\right) \end{array}$$

hold. Finally, the proof of $K\left(\langle \tau_{ua}\left(G_{uac}\right)\rangle \right)+O\left(1\right)\geq K\left(\langle E_{ua}\left(G_{uac}\right)\rangle \;|\;x^{\prime }\right)$ follows directly from Lemma 2. □

For the purpose of comparison, the next immediate question arises from whether there might be such a worst-case distortions between composite edge set strings and characteristic strings when the multidimensional space is *uniform* and the network is *node-aligned*. As the reader might expect, we show in Lemma 5 below that node-aligned MAGs with uniform multidimensional spaces are more tightly associated to their characteristic strings in terms of the algorithmic information and, thus, they cannot display the same distortions as in Theorems 1, 3, and 4. In particular, the distortions in the *node-aligned uniform* case can only grow up to a *logarithmic* term of the order *p*; and this algorithmic information necessary to compute the value of *p* can only grow up to a double logarithmic term of the length of the node-aligned characteristic string:

**Lemma** **5.** *Let $G_{c}$ be an arbitrary node-aligned classical MAG with arbitrarily large uniform multidimensional spaces, where $\left|V\left(G_{c}\right)\right|\geq 3$ and $\left|A\left(G_{c}\right)\left[i\right]\right|\geq 2$ for every i≤p. Then,*

$$\begin{array}{c} K\left(x\right)\leq K\left(\langle E(G_{c})\rangle \right)+O\left(1\right)\leq K\left(x\right)+O\left(\log_{2}\left(p\right)\right)\leq K\left(x\right)+O\left(\log_{2}\left(\log_{2}\left(l(x)\right)\right)\right)\;, \end{array}$$

*where x is the respective node-aligned characteristic string and p is the order of $G_{c}$.*

**Proof.**  Since the multidimensional space is uniform, there is a simple algorithm that always compute the integer value $\left|A\left(G_{c}\right)\left[i\right]\right|$, for any i≤p, when ${✕}_{i=1}^{p}\left|A\left(G_{c}\right)\left[i\right]\right|$ and *p* are given as inputs. In addition, in this case, $\langle \tau (G_{c})\rangle$ can be computably built if $\left|A\left(G_{c}\right)\left[i\right]\right|$, for any i≤p, and the value of *p* are given as inputs. Moreover, by solving a simple quadratic equation with just one possible positive integer solution, there also is a simple algorithm that always returns the integer value ${✕}_{i=1}^{p}\left|A\left(G_{c}\right)\left[i\right]\right|$ when $l\left(x\right)=\left|E_{c}\left(G_{c}\right)\right|$ is given as input. We have from (Lemma 2, p. 6, [15]) that $\langle E(G_{c})\rangle$ can always be computed if $x^{*}$ and $\langle \tau (G_{c})\rangle$ are given as inputs. Thus, by combining all of these algorithms, we will have that

(12) $$\begin{array}{c} K\left(\langle E(G_{c})\rangle \right)\leq K\left(x\right)+O\left(\log_{2}\left(p\right)\right) \end{array}$$

holds. Since $\left|V\left(G_{c}\right)\right|={\left|A\left(G_{c}\right)\left[1\right]\right|}^{p}\;,$ and $\left|A\left(G_{c}\right)\left[1\right]\right|\geq 2$, then we also have that $p\leq \log_{2}\left(\left|V\left(G_{c}\right)\right|\right)\leq \log_{2}\left(l(x)\right)\;.$ Therefore, from Equation (12),

$$K\left(\langle E(G_{c})\rangle \right)\leq K\left(x\right)+O\left(\log_{2}\left(p\right)\right)\leq K\left(x\right)+O\left(\log_{2}\left(\log_{2}\left(l(x)\right)\right)\right)$$

holds. Finally, to prove that $K\left(x\right)\leq K\left(\langle E(G_{c})\rangle \right)+O\left(1\right)$, just note that one can always computably extract *x* from $\langle E(G_{c})\rangle$. □

An interesting future research is to investigate whether one can construct an worst-case example of node-aligned multidimensional network with uniform multidimensional space that actually displays a distortion of the tight order of $\log_{2}\left(p\right)$. In any event, Lemma 5 already demonstrates an *upper bound* for the worst-case distortion increasing rate with respect to the value of *p* (i.e., with the number of node dimensions). In particular, as mentioned before, this upper bound is given by only a logarithmic term of *p*.

On the other hand, although we saw in Lemma 5 that *uniform* multidimensional spaces can only display very small distortions in the *node-aligned* case, we show below in Theorem 4 that worst-case distortions that grow exponentially with *p* are still possible in the *node-unaligned* case. An extended version of the proof of Theorem 4 can be found in [16].

**Theorem** **4.** *There are encodable node-unaligned simple MAGs $G_{uac}$ given $\tau_{ua}\left(G_{uac}\right)$ with $\left|A\left(G_{uac}\right)\left[i\right]\right|\geq 2$ for every i≤p and with arbitrarily large uniform multidimensional spaces such that*

$$K\left(\langle \tau_{ua}\left(G_{uac}\right)\rangle \right)+O\left(1\right)\geq K\left(\langle E_{ua}\left(G_{uac}\right)\rangle \;|\;x^{\prime }\right)\geq K\left(\langle \tau_{ua}\left(G_{uac}\right)\rangle \right)-O\left(\log_{2}\left(K(\langle \tau_{ua}\left(G_{uac}\right)\rangle )\right)\right)$$

*with*

$$\log_{2}\left(K\left(\langle E_{ua}\left(G_{uac}\right)\rangle \right)\right)=\Omega \left(p\right)$$

*and*

$$K\left(x^{\prime }\right)=O\left(\log_{2}\left(K\left(\langle E_{ua}\left(G_{uac}\right)\rangle \right)\right)\right)\;,$$

*where $x^{\prime }$ is the respective node-unaligned characteristic string and p is the order of $G_{uac}$.*

**Proof.**  The underlying idea of this proof is similar to the proof of Theorem 3, but with the fundamental distinction in the set of all composite vertices ${✕}_{i=1}^{p}\left|A\left(G_{uac}\right)\left[i\right]\right|=\left|V\left(G_{uac}\right)\right|$, so that now $\left|V\left(G_{uac}\right)\right|={\left|A\left(G_{uac}\right)\left[1\right]\right|}^{p}$ holds instead of $\left|V\left(G_{uac}\right)\right|=2^{\left(\frac{p}{2}\pm o\left(p\right)\right)}$. Since $\left|A\left(G_{uac}\right)\left[1\right]\right|=\left|A\left(G_{uac}\right)\left[i\right]\right|\geq 2$ for every *i*, then we now have that

(13) $$p\leq p\;\log_{2}\left(\left|A\left(G_{uac}\right)\left[1\right]\right|\right)=\log_{2}\left(\left|V\left(G_{uac}\right)\right|\right)\;.$$

Thus, from the Borel normality of $w_{2}$, there will be a program of length

$$\leq O\left(p\;\log_{2}\left(\left|A\left(G_{uac}\right)\left[1\right]\right|\right)\right)+O\left(\log_{2}\left(p\right)\right)$$

that returns the integer value $\left|E_{uac}\left(G_{uac}\right)\right|$ as output, where $\left|E_{uac}\left(G_{uac}\right)\right|=l\left(x^{\prime }\right)$. Hereafter, the rest of the proof follows analogously to the proof of Theorem 3. □

The proof of Corollary 2 follows from Theorems 2, 3 and 4 in a totally analogous manner as Corollary 1 follows from (Theorem 1, p. 54, [17]) and (Theorem 2, p. 7, [15]). Thus, we choose to leave the following proof up to the reader.

**Corollary** **2.** *There are an infinite family $F_{1}^{\prime }$ of node-unaligned simple MAGs, which may have either uniform or non-uniform multidimensional spaces, and an infinite family $F_{2}^{\prime }$ of classical graphs, where every classical graph in $F_{2}^{\prime }$ is unaligning MAG-graph-isomorphic to at least one MAG in $F_{1}^{\prime }$, such that, for every constant c∈N, there are $G_{uac}\in F_{1}^{\prime }$ and a $G_{G_{uac}}^{ua}\in F_{2}^{\prime }$ that is unaligning MAG-graph-isomorphic to $G_{uac}$, where*

$$O\left(\log_{2}\left(K\left(\langle E_{ua}\left(G_{uac}\right)\rangle \right)\right)\right)>c+K\left(\langle E\left(G_{G_{uac}}^{ua}\right)\rangle \right).$$

Besides showing that node-unaligned multidimensional networks can display exponentially larger algorithmic information distortions with respect to their isomorphic monoplex networks, Theorems 3 and 4 together with Corollary 2 show that these distorted values of algorithmic information content grows *at least* exponentially with the order *p* (i.e., with number of node dimensions).

Finally, we can combine our results in order to achieve our last theorem, which summarizes the results of the present article:

**Theorem** **5.**

(I) *There are an infinite family $F_{1}^{\prime \prime }$ of simple MAGs and an infinite family $F_{2}^{\prime \prime }$ of classical graphs, where every classical graph in $F_{2}^{\prime \prime }$ is MAG-graph-isomorphic to at least one MAG in $F_{1}^{\prime \prime }$, such that:*
  (a) *if the simple MAGs in $F_{1}^{\prime \prime }$ are node-aligned and have a non-uniform multidimensional space, then for every constant c∈N, there are $G_{c}\in F_{1}^{\prime \prime }$ and a $G_{G_{c}}\in F_{2}^{\prime \prime }$ that is (aligning) MAG-graph-isomorphic to $G_{c}$, where*
    $$O\left(\log_{2}\left(K(\langle E(G_{c})\rangle )\right)\right)>c+K\left(\langle E\left(G_{G_{c}}\right)\rangle \right)\;,$$
    *and this exponential distortion grows at least linearly with the order p of the MAG $G_{c}$, that is,*
    $$K\left(\langle E(G_{c})\rangle \right)\geq p-O\left(1\right)\;.$$
  (b) *if the simple MAGs in $F_{1}^{\prime \prime }$ are* node-unaligned *and have either non-uniform or uniform multidimensional spaces, then, for every constant c∈N, there are $G_{uac}\in F_{1}^{\prime \prime }$ and a $G_{G_{uac}}^{ua}\in F_{2}^{\prime \prime }$ that is (unaligning) MAG-graph-isomorphic to $G_{uac}$, where*
    $$O\left(\log_{2}\left(K\left(\langle E_{ua}\left(G_{uac}\right)\rangle \right)\right)\right)>c+K\left(\langle E\left(G_{G_{uac}}^{ua}\right)\rangle \right)\;,$$
    *and this exponential distortion grows at least exponentially with the order p of the MAG $G_{uac}$, that is,*
    $$\log_{2}\left(K\left(\langle E_{ua}\left(G_{uac}\right)\rangle \right)\right)=\Omega \left(p\right)\;.$$
(II) *Let $F_{1}^{\prime \prime }$ be an arbitrary infinite family of node-aligned classical MAGs with* uniform *multidimensional spaces. Let $F_{2}^{\prime \prime }$ be an arbitrary infinite family of classical graphs such that every classical graph in $F_{2}^{\prime \prime }$ is (aligning) MAG-graph-isomorphic to at least one MAG in $F_{1}^{\prime \prime }$ and that both these graph and MAG share the same characteristic string. Then, for every $G_{c}\in F_{1}^{\prime \prime }$ and $G_{G_{c}}\in F_{2}^{\prime \prime }$ that is (aligning) MAG-graph-isomorphic to $G_{c}$, one has that*
  $$K\left(\langle E\left(G_{G_{c}}\right)\rangle \right)\leq K\left(\langle E(G_{c})\rangle \right)+O\left(1\right)\leq K\left(\langle E\left(G_{G_{c}}\right)\rangle \right)+O\left(\log_{2}\left(p\right)\right)$$
  *and, therefore, any distortion can only grow up to a logarithmic order of p.*

**Proof.**  The proof of Theorem 5(I)(a) follows directly from Theorem 1 and Corollary 1, which were previously demonstrated in [15]. The proof of Theorem 5(I)(b) follows directly from Theorems 3 and 4 together with Corollary 2. The proof of Theorem 5(II) follows directly from Lemma 5 and (Lemma 2, p. 6, [15]) by fixing a computable ordering for both the sets $E_{c}\left(G_{c}\right)$ and $E_{c}\left(G_{G_{c}}\right)$. □

## 5. Limitations and Conditions for Importing Monoplex Network Algorithmic Information to Multidimensional Network Algorithmic Information

It is known that applying statistical informational measures, such as those based on entropy, to evaluate lossless compressibility or irreducible information content of encoded objects may lead to deceiving values in some cases. For example, for some particular low-algorithmic-complexity networks that display maximal degree-sequence entropy [29] or for Borel-normal sequences that are in fact computable (and, therefore, logarithmically compressible) [30], distortions between the entropy-based lossless compression and the algorithmic-information-based approximation can occur. Other discrepancies between entropy and algorithmic complexity in network models can be found in [6].

In this regard, one of the advantages of algorithmic information is that, at least in the asymptotic limit when the computational resources are unbounded, the lossless compression is proven to be optimal. In addition, it gives values that are object invariant. This is because, given any two distinct encoding methods or any two distinct universal programming languages, the algorithmic complexity of an encoded object represented in one way or the other can only differ by a constant whose value only depends on the choice of encoding methods or universal programming languages, and not on the encoded object being compressed. That is, algorithmic complexity is pairwise invariant for any two arbitrarily chosen encodings.

The present article contributes by showing that, even in the asymptotically optimal case given by the algorithmic information, distorted values can occur in multidimensional networks with sufficiently large multidimensional spaces. Although only dealing with pairs of isomorphic objects in addition to the above encoding invariance, some may deem the existence of the distortion phenomena shown in the present work as counter-intuitive at first glance because they can, in fact, result from only changing the multidimensional spaces into which isomorphic copies of the objects are embedded.

In order to avoid these distortions in future evaluations of multidimensional network complexity, our results demonstrate the importance of network representation methods that take into account the algorithmic complexity of the data structure itself, unlike what happens for example with adjacency matrices, tensors, or characteristic strings. One of those representation methods, which was studied in this article, that avoids distortions is using composite edge set strings. Nevertheless, our results hold for any other form of encoding a simple MAG that is Turing equivalent to a composite edge set string. For example, one can equivalently encode a composite edge set string as an three-dimensional array composed of positive integers, Boolean variables, and lists: the first dimension stores the index value $\langle a_{1},\cdots ,a_{p}\rangle$ of each composite vertex $v=\left(a_{1},\cdots ,a_{p}\right)$; the second dimension stores a Boolean value determining whether the composite vertex in the first dimension exists or not; and the third dimension stores a list containing the index value of each composite vertice with which the composite vertex in the first dimension forms a composite edge.

On the other hand, when importing previous algorithmic-information-based methods from monoplex networks (or graphs) into the multidimensional case, another method to deal with the algorithmic information distortions is to accept an error margin given by the algorithmic complexity of the companion tuple. This occurs because our results directly establish that the algorithmic information distortions are always upper bounded by the algorithmic information carried by the companion tuple, whether node-aligned or node-unaligned. Thus, even in the worst-case scenario, the value of the algorithmic complexity of the companion tuple can be always applied as an error margin for the algorithmic information distortions between multidimensional networks and their isomorphic graphs.

## 6. Conclusions

This article presented mathematical results on network complexity, irreducible information content, and lossless compressibility analysis of node-aligned or node-unaligned multidimensional networks. We studied the limitations for algorithmic information theory (AIT) applied to monoplex networks or graphs to be imported into multidimensional networks, in particular, in the case the number of extra node dimensions (i.e., aspects) in these networks is sufficiently large. Our results demonstrate the existence of worst-case algorithmic information distortions when a multidimensional network is compared with its isomorphic monoplex network. More specifically, our proofs show that these distortions exist when a logarithmically compressible network topology of a monoplex network is embedded into a high-algorithmic-complexity multidimensional space.

Previous results in [15] have shown that *node-aligned* multidimensional networks with *non-uniform* multidimensional spaces can display an exponentially larger algorithmic complexity than the algorithmic complexity of their isomorphic monoplex networks. In addition, Abrahão et al. [15] show that these distorted values of algorithmic information content grow at least linearly with number of extra node dimensions.

When dealing with either *uniform* or *non-uniform* multidimensional spaces, we show in this article that *node-unaligned* multidimensional networks can also display exponential algorithmic information distortions with respect to the algorithmic information content of their respective isomorphic monoplex networks. Unlike the case studied in [15], these worst-case distortions in the node-unaligned case are shown to grow at least exponentially with the number of extra node dimensions. Thus, the node-unaligned case is more impactful than the previous node-aligned one precisely because exponential distortions may take place even with uniform multidimensional spaces. Furthermore, the distortions may grow much faster as the number of extra node dimensions increases.

On the other hand, we demonstrated that *node-aligned* multidimensional networks with *uniform* multidimensional spaces are limited to only displaying algorithmic information distortions that grow up to a logarithmic order of the number of extra node dimensions. As one might expect, the node alignment in conjunction with the uniformity of the multidimensional space guarantee that, in any event, the algorithmic information content of the multidimensional network and the algorithmic information content of its isomorphic monoplex network are tightly associated, except maybe for a logarithmic factor of the number of extra node dimensions.

The results in this article show that evaluations of the algorithmic information content of networks may be extremely sensitive to whether or not one is taking into account not only the total number of node dimensions but also the respective sizes of each node dimension, and the order that they appear in the mathematical representation. Due to the need for additional irreducible information in order to compute the shape of the high-algorithmic-complexity multidimensional space, the present article shows that isomorphisms between finite multidimensional networks and finite monoplex networks do not preserve algorithmic information in general, so that the irreducible information content of a multidimensional network may be highly dependent on the choice of its encoded isomorphic copy. In order to avoid distortions in the general case when studying network complexity or lossless compression of multidimensional networks, it also highlights the importance of embedding the information necessary to determine the multidimensional space itself into the encoding of the multidimensional network. To such an end, network representation methods that take into account the algorithmic complexity of the data structure itself (unlike adjacency matrices, tensors, or characteristic strings) are required for importing algorithmic-information-based methods into the multidimensional case. In this way, given the relevance of algorithmic information theory in the challenge of causality discovery in network modeling, network summarization, network entropy, and compressibility analysis of networks, we believe this paper makes a fundamental contribution to the study of the complexity of multidimensional networks that have a large number of node dimensions, which in turn also imposes a need to be accompanied by more sophisticated algorithmic complexity approximating methods than those for monoplex networks or graphs.

## Data Availability

Not applicable.
